# BCG revaccination qualitatively and quantitatively enhances SARS-CoV-2 spike-specific neutralizing antibody and T cell responses induced by the COVISHIELD^™^ vaccine in SARS-CoV-2 seronegative young Indian adults

**DOI:** 10.21203/rs.3.rs-1395683/v1

**Published:** 2022-03-02

**Authors:** Srabanti Rakshit, Vasista Adiga, Asma Ahmed, Chaitra Parthiban, Nirutha Chetan Kumar, Pratibha Dwarkanath, Sudarshan Shivalingaiah, Srishti Rao, George D’Souza, Mary Dias, Thomas J.A. Maguire, Katie Doores, Prokar Dasgupta, Sudhir Babji, Tom H.M Ottenhoff, Kenneth D. Stuart, Stephen De Rosa, M. Juliana McElrath, Annapurna Vyakarnam

**Affiliations:** 1 Centre for Infectious Disease Research, Indian Institute of Science, Bangalore, India; 2 Infectious Disease Unit, St. John’s Research Institute, Bangalore, India; 3 Division of Nutrition, St. John’s Research Institute, Bangalore, India; 4 Department of Pulmonary Medicine, St. John’s Medical College, Bangalore, India; 5 Department of Infectious Diseases, School of Immunology and Microbial Sciences, King’s College London; 6 Peter Gorer Department of Immunobiology, Liver Renal Urology Transplant Gastro/Gastrointestinal Surgery, Inflammation Biology, King’s College, London; 7 The Wellcome Trust Research Laboratory, Christian Medical College, Vellore, India; 8 Dept. Infectious Diseases, Leiden University Medical Center, Leiden, The Netherlands; 9 Seattle Children’s Research Institute, Seattle, Washington USA; 10 Vaccine and Infectious Disease Division, Fred Hutchinson Cancer Research Centre, Seattle, Washington, USA; 11 Department of Medicine, University of Washington School of Medicine, Seattle, Washington, USA; 12 Department of Immunobiology, School of Immunology & Microbial Sciences, Faculty of Life Science & Medicine, King’s College, London

**Keywords:** BCG, SARS-CoV-2, COVISHIELD^™^, CD4, CD8, antibody binding, neutralizing antibody, heterologous responses, trained immunity

## Abstract

This study tested if prior BCG revaccination can further boost immune responses subsequently induced by a widely distributed and otherwise efficacious Oxford/AstraZeneca ChAdOx1nCoV-19 vaccine, referred to as COVISHIELD^™^, in India. We compared COVISHIELD^™^ induced longitudinal immune responses in 21 BCG re-vaccinees (BCG-RV) and 13 BCG-non-revaccinees (BCG-NRV), all of whom were BCG vaccinated at birth and latent tuberculosis negative, after COVISHIELD^™^ prime and boost with baseline samples that were collected pre-pandemic and pre-BCG revaccination. Compared to BCG-NRV, BCG-RV displayed significantly higher magnitude of spike-specific Ab and T cell responses, including a greater proportion of high responders; better quality polyfunctional CD4 and CD8 T cells that persisted and a more robust Ab and T cell response to the Delta mutant of SARS-CoV-2 highlighting greater breadth. Mechanistically, BCG adjuvant effects on COVISHIELD^™^ induced adaptive responses was associated with more robust innate responses to pathogen-associated-molecular-patterns through TNF-α and IL-1β secretion. This study provides first in-depth analysis of immune responses induced by COVISHIELD^™^ in India and highlights the potential of using a cheap and globally available vaccine, BCG, as an adjuvant to enhance heterologous adaptive immune responses induced by COVIDSHIELD^™^ and other emerging vaccines.

## INTRODUCTION

Bacille Calmette-Guérin (BCG) vaccine is part of vaccination policy at birth for tuberculosis (TB) prevention in several TB endemic countries^1^. Beyond TB, heterologous benefits of BCG include reducing all-cause mortality and morbidity in infants and children^2,3^, with these non-specific effects providing cross-protection against other pathogens, particularly against viral respiratory infections^4–7^. The heterologous benefits of BCG vaccination are likely mediated via the combined induction of Th1/Th17, humoral and innate immune responses. Increasing evidence emphasize that BCG vaccination enhances innate immune memory or ‘trained immunity’ (TI)^8,9^, a biological process by which innate immune response to pathogen-associated molecular patterns (PAMP) is significantly amplified by prior BCG exposure^10^. BCG induced trained immunity is mediated through epigenetic reprogramming and cellular metabolism rewiring^11,12^, an imprint that can be retained, such that subsequent PAMP exposure induces more pronounced innate effector function in monocytes^13,14^, This, in-turn contributes to an immune response that can be more protective than the one generated in the absence of prior BCG exposure^10,15–17^.

The COVID-19 outbreak has refocused interest in the cross-protective benefits of BCG. Despite effective vaccines^18^, COVID-19 remains a global concern due to emergence of newer variants and global vaccine inequity. Consequently, more widely available vaccines such as the Influenza, OPV, MMR, Varicella Zoster and BCG vaccines known to boost heterologous cross-protective immunity are currently being tested for their efficacy against SARS-CoV-2 in various clinical trials^19–23^, with the most compelling evidence for BCG emerging from murine studies^24,25^. The intravenous administration of BCG in human-ACE2 transgenic mice followed by lethal SARS-CoV-2 challenge reduced viral load, resulted in milder disease and increased survival^24^. However, a similar study in rhesus macaques found no evidence of aerosol BCG vaccination protecting against SARS-CoV-2 challenge^26^. Whilst comprehensive data of BCG impact on COVID-19 infection and /or disease severity is awaited from global human trials^27,28^, several lines of evidence highlight potential benefit. Mathematical modelling highlighted administration of vaccines with heterologous benefits including BCG even with 5–15% efficacy could reduce COVID-19 cases, hospitalization, and mortality in the United States^29^. Epidemiological studies show countries with mandatory BCG vaccination recorded a lower incidence of SARS-CoV-2 infection and COVID-19 related deaths compared to countries where routine immunization was absent or terminated^30–36^. Individuals vaccinated with BCG in the past five years reported lower incidence of illness and fatigue compared to BCG non-vaccinated subjects^37^ and anti-SARS-CoV-2 IgG seroprevalence and self-reported COVID-19 symptoms were significantly less in healthcare workers with BCG vaccination history^38^. In contrast, other studies do not report similar benefits^39,40^ perhaps due to the timing of BCG vaccination prior to SARS-CoV-2 exposure or the BCG strain used^41–43^.

Beyond improving immunity to natural infections, BCG can enhance adaptive responses to heterologous vaccines in adults and infants^44–48^. Antibody levels against Influenza A (H1N1) were boosted and seroconversion accelerated in individuals who received BCG prior to a trivalent influenza vaccine^44^. Humoral responses to vaccination against pneumococcus, tetanus toxoid, measles, mumps, diphtheria, polio were higher in BCG immunized compared to non-BCG immunized infants in several trials^46–48^. Importantly, data from a randomized study in Mexico showed neutralizing antibody (NAb) titers induced by the Pfizer–BioNTech SARS-CoV-2 vaccine to be higher in the group that received BCG first and then the SARS-CoV-2 vaccine relative to the group that received placebo and SARS-CoV-2 vaccine^49^. In an animal study, human-ACE2 transgenic mice given BCG coupled with a trimeric-Spike vaccine generated a higher titer of NAb and a greater Th1 response than controls receiving trimeric–Spike vaccine alone and cleared infection with minimal immunopathology following SARS-CoV-2 challenge^25^. However, prior exposure to BCG does not guarantee an enhanced adaptive response to all vaccines: Responses to the Vi polysaccharide typhoid fever vaccine (TFV) and the *Haemophilus influenzae* type B (anti-Hib) vaccine was not boosted upon prior BCG exposure^45,47,48^. Individual vaccines, whether they are live attenuated, whole inactivated or subunit vaccines, can impact the immune system in various ways, and this may or may not necessarily synergize with the non-specific effects of BCG.

These considerations prompted us to explore the impact of prior BCG vaccination on immune responses induced by the Oxford-AstraZeneca ChAdOx1-S, the first SARS-CoV-2 vaccine to be rolled out in India, locally referred to as COVISHIELD^™^ manufactured by the Serum Institute of India. Our study was exceptionally well positioned to address the above objective: we had initiated a BCG revaccination study of young healthcare workers in October 2019 residing at St. John’s Medical College Hospital, Bangalore with baseline samples (prior to BCG revaccination) and samples at 1 day and 8–10 weeks post BCG revaccination collected before the first COVID-19 outbreak in India. We were therefore in a unique position to track these subjects’ longitudinal responses to the COVISHIELD^™^ vaccine rolled out in January 2021 relative to their pre-pandemic baseline samples. Our study is the first to provide novel insights into the magnitude and quality of antibody and T cell responses elicited by the COVISHIELD^™^ vaccine in young Indian adults, who were seronegative against all SARS-CoV-2 proteins screened. This entitles unequivocal analysis of the primary immune response induced by COVISHIELD^™^ prime vaccination to be placed in context of the COVISHIELD^™^ booster dose in subjects who did and did not receive prior BCG revaccination.

## RESULTS

### Study Overview (Figure 1).

**Figure 1:**
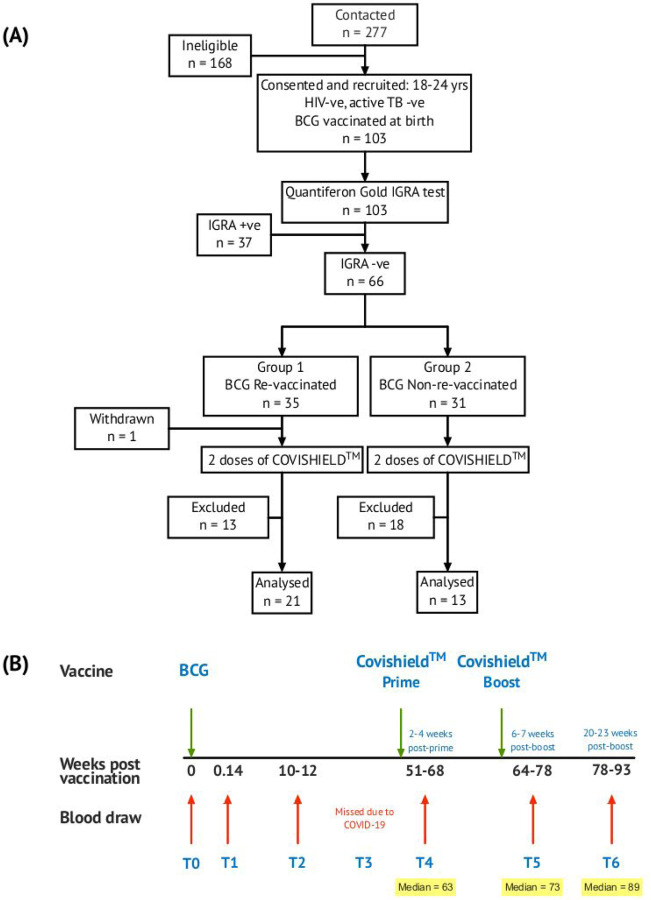
Overall study design. **(A)** CONSORT flow diagram of participant recruitment and enrolment. **(B)** A diagrammatic representation of study design, including schedule of BCG and COVISHIELD^™^ vaccination and blood draw. Group 1 received BCG at day 0 (T0) and then both groups were vaccinated with 2 doses of COVISHIELD^™^ vaccine (Prime and Boost). Time points for immunization with BCG and COVISHIELD^™^ are shown by green arrows, and the 6 blood sampling time points (T0–T6) are indicated by red arrows for all groups.

Funded in 2019, this study, was initially designed to probe the efficacy of BCG revaccination on enhancing *Mycobacterium tuberculosis* (Mtb)-specific immune responses, following our previous work^50^. Due to the COVID-19 pandemic, we pivoted to probe the impact of BCG revaccination on COVISHIELD^™^ vaccine-induced immune responses. We recruited 103 young health care workers at St. John’s Medical College Hospital who had all received BCG vaccine at birth, of whom 66 were confirmed Interferon Gamma Release Assay (IGRA) negative (Table 1). Of these, 35 received BCG revaccination and 31 did not (see Materials and Methods). Samples were collected at baseline (T0), 1 day (T1) and 8–10 weeks -post BCG revaccination (T2); these time points were between September 2019 and January 2020 before COVID-19 pandemic spread to India. Subsequent sample collection (T3) between March 2020 and August 2020 was abandoned. Following roll out of the COVISHIELD^™^ vaccine in January 2021, we collected samples post COVISHIELD^™^ vaccination from both BCG re-vaccinees (BCG-RV) and BCG non-vaccinees (BCG-NRV). The median interval between BCG revaccination and the first dose of COVISHIELD^™^ vaccine was 63 weeks. We collected samples at 2-, 3- or 4-weeks after COVISHIELD^™^ prime (collectively referred to as T4 prime time point), followed by 5–6 weeks after the booster vaccine (T5 time point), or at 20–23 weeks post-booster (T6 time point). Due to COVID-19 restrictions, subjects were sampled only at one time point post prime and one time point post boost. For COVISHIELD^™^ vaccine-induced immune data analysis, the following samples were excluded: (i) samples not matched to the above time points, (ii) all subjects who tested SARS-CoV-2 RT-PCR positive, (iii) subjects identified to be potentially exposed to SARS-CoV-2 based on a positive in-house IgG antibody binding assay to the SARS-CoV-2 nucleocapsid (N) protein (see Extended Data Fig. 1), and (iv) all dropout subjects where we were unable to collect samples at one of the prime and one of the boost time points. In total, we successfully collected time matched samples from 34 subjects, of which 21 were BCG Re-vaccinees (referred to as BCG-RV) and 13 were not re-vaccinated with BCG (referred to as BCG-NRV) (Fig. 1).

### Overview of COVISHIELD^™^ induced spike-specific immune responses in young Indian adults.

**Figure 2:**
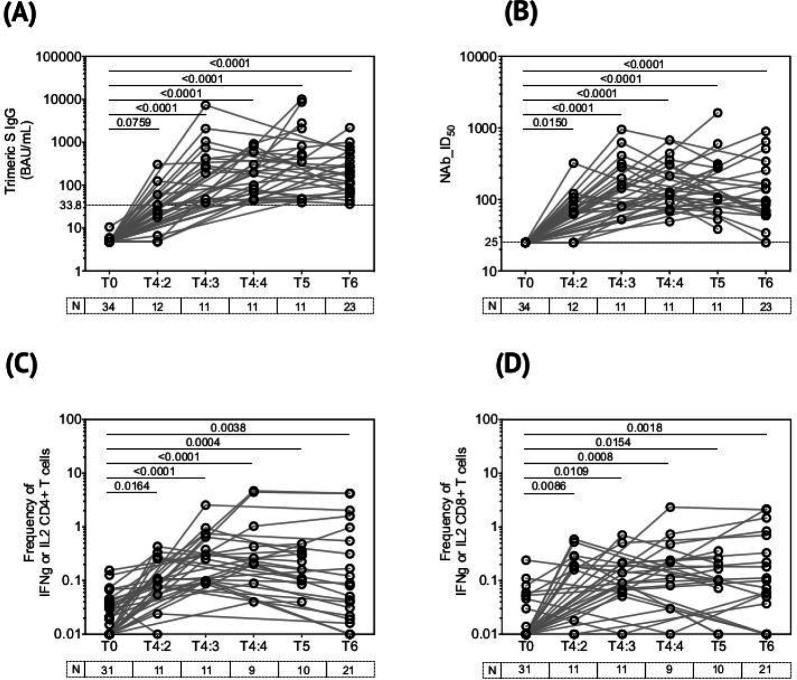
Overview of spike-specific immune responses to COVISHIELD^™^ vaccination and the impact of time. **(A)** Plasma SARS-CoV-2 anti-spike protein IgG titres by LIAISON® SARS-CoV-2 TrimericS IgG assay and **(B)** Neutralizing antibody responses (NAb ID_50_) in COVISHIELD^™^ vaccinated subjects measured at baseline (T0), 2-, 3- and 4-weeks post-prime (T4:2, T4:3 and T4:4), 6–7 weeks post boost (T5) and 20–23 weeks post-boost (T6). **(C & D)** CD4+ and CD8+ T cell responses in COVISHIELD^™^ vaccinated subjects measured overtime. PBMCs from individuals collected at baseline (T0), 2-, 3- and 4-weeks post-prime (T4:2, T4:3 and T4:4), 6–7 weeks post boost (T5) and 20–23 weeks post-boost (T6) were stimulated with Spike peptide pool (0.06 nM) for 20 hr. CD4+ and CD8+ T cells were analyzed for intracellular expression of IFN-γ or IL-2. Kruskal-Wallis test with Dunn’s correction was used for determining statistical significance.

We used four immune assays to track COVISHIELD^™^ vaccine induced responses in all 34 subjects recruited: antibody-binding LIAISON® SARS-CoV-2 TrimericS IgG assay (Fig. 2A); NAb (ID_50_) titres (Fig. 2B); spike-specific frequencies of IFN-γ or IL-2 expressing CD4+ (Fig. 2C) and CD8+ (Fig. 2D) T cells measured by intracellular cytokine staining (ICS) assay for which representative gating is shown in Extended Data Fig. 2. Statistically significant (P<0.05) induction of Ab and T cell responses was observed at 3 and 4 weeks post priming (T4:3 and T4:4) and remained significantly higher than baseline after the booster (T5 and T6). Ab binding and NAb peaked at 3 weeks, dipping at 4 weeks; lowest Ab responses were detected at 2 weeks post-prime. CD4+ T cell response also peaked at 3 weeks and sustained up to 4 weeks post prime, whilst peak CD8+ T cell responses were noted at 4 weeks, with some individuals responding exceptionally well as early as 2 weeks post prime. Analysis of the booster vaccine effect revealed a clear increase in both Ab binding and NAb concentrations at 5–6 weeks post booster relative to the 4 week post prime levels; however, a similar increase was not noted for CD4+ or CD8+ T cell frequencies in majority of donors tested. We report >87% vaccine potency; thus, subjects who did not respond to both prime and boost was 0% for Ab binding (Fig. 2A, 0/34); 2.9% for NAb (Fig. 2B 1/34); 3.2% for CD4+ T cell responses (Fig. 2C, 1/31) and 12.9% for CD8+ T cell responses (Fig. 2D, 4/31) as far as we could measure. Of the responders, the percentage who required a booster to respond was variable: this was 20.6% for Ab binding (Fig. 2A, 7/34) using 33.8BAU/ml cut-off, which may be high as only 3 of the same subjects were detected as needed a boost by NAB assay (9.1% for Nab, Fig. 2B, 3/33); 0% for CD4+ T cell responses (Fig. 2C, 0/30) and 14.8% for CD8+ T cell responses (Fig. 2D, 4/27).

Taken together, these data highlight the COVISHIELD^™^ vaccine to be highly efficient in inducing immune responses in young Indian adults, with priming alone inducing significant Ab and T cell responses. The booster vaccine effect is particularly noticeable in Ab responses, consistent with other studies^51–55^. In contrast, the highest responses noted for CD4+ and CD8+ T cells was at 3–4 weeks post prime, also consistent with other reports^51–55^. Both Ab and T cell responses waned by 20–23 weeks; importantly, in vast majority of subjects the induced responses did not fall to baseline.

### BCG revaccination enhances the magnitude of COVISHIELD^™^ induced spike-specific immune responses.

**Figure 3:**
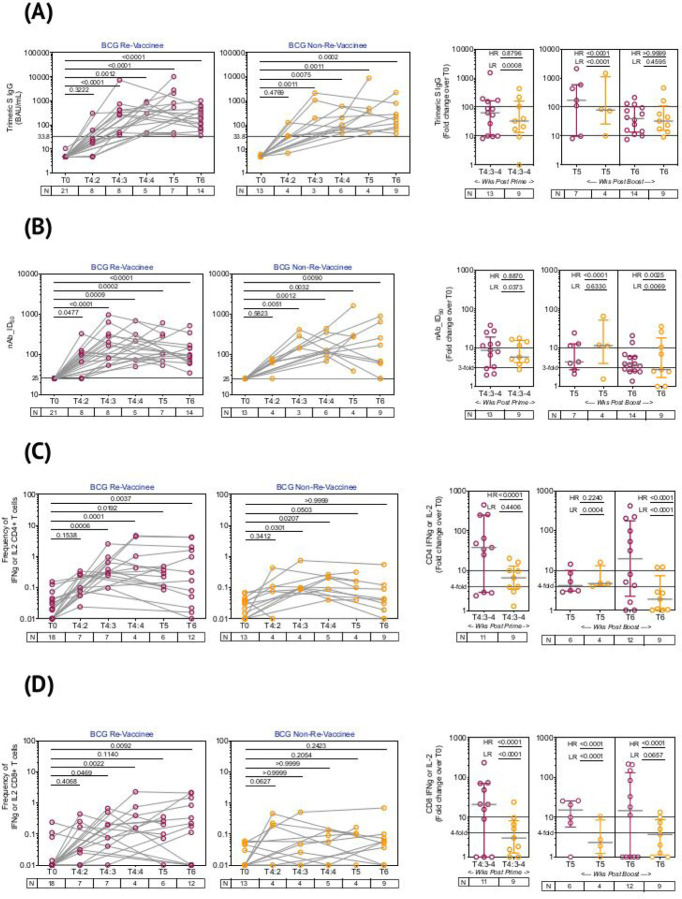
Overview of spike-specific vaccine induced responses in BCG-RV and BCG-NRV. **(A)** Plasma SARS-CoV-2 anti Spike protein IgG titres by LIAISON® SARS-CoV-2 TrimericS IgG assay and **(B)** Neutralizing antibody titres (NAb ID_50_) in COVISHIELD^™^ vaccinated BCG-RV (purple circles) and BCG-NRV (orange circles) subjects measured at baseline (T0), 2-, 3- and 4-weeks post-prime (T4:2, T4:3 and T4:4), 6–7 weeks post boost (T5) and 20–23 weeks post-boost (T6). Grouped scatter plot with median (horizontal grey line) and interquartile range comparing fold change over baseline at 3–4 weeks post-prime (T4:3–4), 6–7 weeks post boost (T5) and 20–23 weeks post boost (T6). **(C & D)** CD4+ and CD8+ T cell responses in COVISHIELD^™^ vaccinated subjects measured overtime. PBMCs from individuals collected from COVISHIELD^™^ vaccinated BCG-RV (purple circles) and BCG-NRV (orange circles) at baseline (T0), 2-, 3- and 4-weeks post-prime (T4:2, T4:3 and T4:4), 6–7 weeks post boost (T5) and 20–23 weeks post-boost (T6) were stimulated with Spike peptide pool (0.06 nM) for 20 hr. CD4+ and CD8+ T cells were analyzed for intracellular expression of IFN-γ or IL-2. Grouped scatter plot with median (horizontal grey line) and interquartile range comparing the fold change of IFN-γ or IL-2 in (C) CD4+ and (D) CD8+ T cells collected at 3–4 weeks post-prime (T4:3–4), 6–7 weeks post boost (T5) and 20–23 weeks post boost (T6). Kruskal-Wallis test with Dunn’s correction was used for determining statistical significance between longitudinal timepoints. The proportion of each group that showed a positive serologic response to Spike, neutralizing antibody titres or a positive IFN-γ or IL-2 CD4+ and CD8+ T cell response to Spike were compared between COVISHIELD^™^ vaccinated BCG-RV (purple circles) and BCG-NRV (orange circles) by using Fisher’s exact test. HR indicates the p-value for high-responders in each group (subjects with >100-fold change over baseline for TrimericS IgG, >10-fold change for NAb, CD4+ or CD8+ T-cell responses). LR indicates the p-value for low-responders (>10-fold change for TrimericS IgG, >3-fold change for NAb and >4-fold change for CD4 or CD8 T-cell responses).

The line graphs in Figure 3 demonstrate the time course of COVISHIELD^™^ induced Spike specific Ab binding, NAb (Fig. 3A and 3B respectively) and CD4+ and CD8+ T cell responses (Fig. 3C and 3D respectively) in BCG-RV and BCG-NRV groups. The data highlight three points: (i) the kinetics were similar in BCG-RV and BCG-NRV: Ab responses peaked at 3 weeks, dipped at 4 weeks post prime and increased at 5–6 weeks post booster with a decline after 20–23 weeks post the booster (Fig. 3A and 3B). (ii) CD4+ T cell responses peaked at 4 weeks post prime in both BCG-RV and BCG-NRV subjects with subsequent decline. CD8+ T cell responses also peaked at 4 weeks post prime noticeable in BCG-RV, with no further increase by the booster vaccine. Instead, a contraction of spike-specific CD4+ and CD8+ T-cell frequencies was noted at both the T5 and T6 time points relative to peak values, with some noticeable differences: median CD4+ and CD8+ T cell frequencies were higher in BCG-RV versus BCG-NRV, with a trend to remain higher in BCG-RV at 20–23 weeks post boost (Fig. 3C and 3D). Furthermore, BCG-NRV CD4+ and CD8+ T cell responses contracted to near baseline levels at T6, whereas in BCG-RV, the median remained 10-fold higher than the baseline. (iii) The proportion of COVISHIELD^™^ non-responders (subjects who did not respond to either prime and boost) did not differ between BCG-RV and BCG-NRV.

Comparative analysis of COVISHIELD^™^ induced responses in BCG-RV and BCG-NRV was conducted by stratifying responders in time matched samples (Fig. 3 scatter graphs) using a contingency Fischer’s Exact Test comparing fold induction of responses, relative to T0. Fold changes were stratified into high and low, dependent on median fold change and spread recorded in each assay: for Ab binding (Fig. 3A) high responses (HR) were set at minimum 100-fold increase over baseline and low responses (LR) set at minimum 10-fold increase over baseline. For NAb (Fig. 3B), HR was 10-fold minimum and LR 3-fold minimum. For CD4+ (Fig. 3C) and CD8+ (Fig. 3D) T cell responses, HR was 10-fold minimum and LR 4-fold minimum. In each immune assay, BCG-RV had a significantly higher proportion of HR and/or LR responders than BCG-NRV (between group comparison minimum p value p=0.0373; maximum p<0.0001). For NAb (Fig. 3B, scatter graph), the biggest difference was noted after the booster at 20–23 weeks. For both CD4+ (Fig. 3C) and CD8+ T cell responses (Fig. 3D), BCG-RV had a greater proportion of high responders after COVISHIELD^™^ priming (T4) and the proportion of high responders persisted up to 20–23 weeks after the booster (T6). Taken together, the data highlights BCG-RV to be significantly better and more often high responders to the COVISHIELD^™^ vaccine than BCG-NRV subjects.

Additionally, we confirm the COVISHIELD^™^ vaccine induced T cell response to be specific. Parallel cultures were tested for T cell stimulation with peptide pools covering SARS-CoV-2 M or N (proteins not included in the COVISHIELD^™^ vaccine), as well as a control recall antigen, CEFT (see methods). Extended Data Fig. 3 demonstrates lack of induction of CD4+ or CD8+ T cell responses to M and N proteins across all samples and no changes to basal CEFT responses over time.

COVISHIELD^™^ induced NAb and T cell responses (Extended Data Fig. 4) showed a significant correlation (*p* range: <0.0069 to <0.0001) that was not strictly linear (r range: 0.4609 to 0.6848) across all samples tested, with correlation significance holding when samples were divided into BCG-RV and BCG-NRV groups. These data confirmed that in both BCG-RV and BCG-NRV subjects, COVISHIELD^™^ vaccination concurrently induced spike-specific Ab and T cell responses.

### BCG revaccination significantly enhances the quality of spike-specific T cell responses.

**Figure 4:**
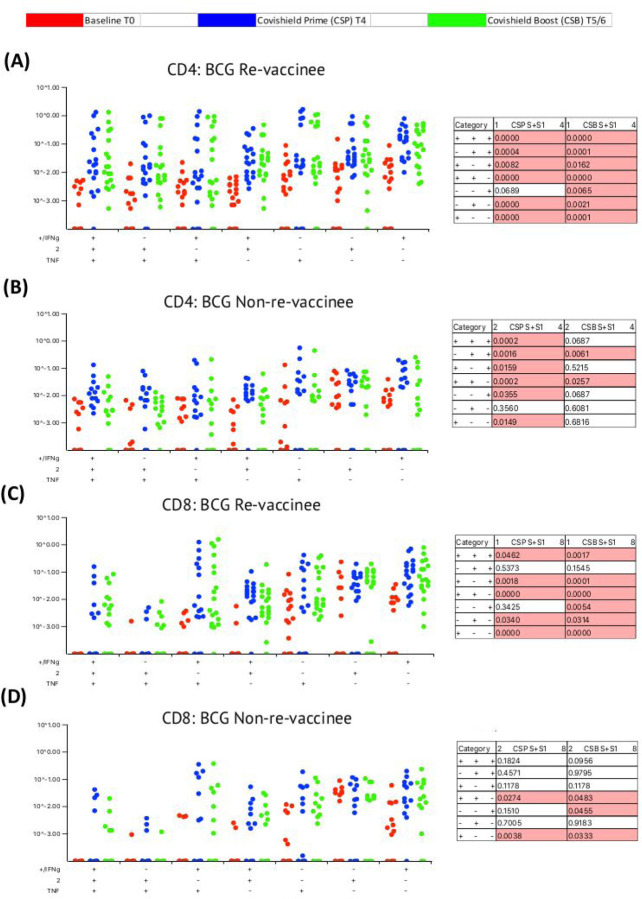
BCG revaccination significantly impacts the quality of the spike-specific CD4+ and CD8+ T cell response in COVISHIELD^™^-vaccinated subjects. Longitudinal multifunctional spike- specific CD4+ T cells **(A&B)** or CD8+ T cells **(C&D)** in COVISHIELD^™^ vaccinees. PBMCs from individuals collected at baseline (BL, red dots), 2–4 weeks post-prime (CSP, blue dots) and 6–7- or 20–23-weeks post-boost (CSB, green dots) were stimulated with spike for 20hr and CD4+ or CD8+ T cells were analyzed for intracellular expression of IFN-γ, IL-2 and TNF-α in a standard ICS assay. Boolean gates were created from the individual cytokines (listed above) in FlowJo to divide responding cells into 7 distinct subsets corresponding to all possible combinations of these functions, and the data were analyzed using SPICE software. Data were analyzed for statistical significance using Wilcoxon signed-rank test. Background subtracted and log data analyzed in all cases. P < 0.05 was considered statistically significant.

Our immune-staining panel included 5 effector cytokines (IFN-γ, IL-2, TNF-α, IL-17 and IL-10), enabling the analysis of cellular subsets expressing combinations of 5, 4, 3, 2 and 1 effectors using SPICE software (https://niaid.github.io/spice/). Initial analysis demonstrated: IL-17 expression in only few subjects, IL-10+ cells rarely detected and robust induction of IFN-γ, IL-2 and TNF-α expressing T cells (see Extended Data Fig. 5); therefore, downstream analysis focussed on effector subsets expressing these three cytokines. Figure 4 shows the pattern of seven CD4+ T cell subsets expressing IFN-γ, IL-2 and TNF-α induced after prime (blue dots) and after booster (green dots), relative to matched baseline value (red dots) in BCG-RV (Fig. 4A) and BCG-NRV samples (Fig. 4B) with each dot representing a donor. The matched table lists p values calculated by SPICE of the induced response over baseline at prime (CSP) and after boost (CSB). BCG-RV significantly induced spike-specific CD4+ T cell subsets expressing 3, 2 and 1 effectors after prime, which were sustained until 20–23 weeks after the booster (Fig. 4A). In BCG-NRV, TNF-α+ and IL-2+ single positive (SP) expressing CD4+ T cells were not induced significantly after prime (Fig 4B), whilst cellular subsets expressing 3, some 2 and some 1 combinations of effectors were. Additionally, in BCG-NRV, only two of the seven subsets analysed (namely: IL-2/TNF-α double positive {DP} and IFN-γ/IL-2 DP) were sustained after the booster: in particular, cells expressing all 3 cytokines and single effectors were not sustained (Fig. 4B, Table). Unpaired t-test with Welch correction of matched BCG-RV and BCG-NRV samples after the prime- (Extended Data Fig. 6A) and booster-vaccines (Extended Data Fig. 6B) showed significantly higher responses in BCG-RV for IFN-γ/IL-2 DP cells and IFN-γ SP after the prime dose and significantly higher IFN-γ/TNF-α DP, TNF-α SP and IFN-γ SP after the booster dose. There was also a trend (p=0.06–0.08) for most other subsets to be expressed higher in BCG-RV after the booster. A similar analysis of CD8+ T cell subsets (Fig. 4C and 4D) highlighted striking differences: whereas five of seven subsets analysed were induced significantly in BCG-RV group at prime and six of seven subsets including 3+ effectors sustained after the booster dose (Fig. 4C, Table), in BCG-NRV five of the seven CD8+ subsets tested and four of seven were not significantly induced after the prime or booster doses, respectively (Fig. 4D). Unpaired t-test with Welch correction of matched BCG-RV and BCG-NRV samples shows no significant differences between the two groups after prime (Extended Data Fig. 6C) or booster (Extended Data Fig. 6D), although, four subsets, namely 3+ IFN-γ+TNF-α+IL-2+ effectors, IFN-γ+/TNF-α+ DP, TNF-α+ SP and IFN-γ+ SP effectors showed a trend (P=0.08) for higher expression in BCG-RV group.

Taken together, these data highlight that BCG revaccination significantly enhances the quality of the COVISHIELD^™^ induced T cell response, with the most pronounced effect being on spike-specific CD4+ rather than CD8+ T cell effectors. To understand if this difference is in part explained by inherent differences in the robustness by which BCG revaccination regulates adaptive CD4+ versus CD8+ T cells, we enumerated BCG-specific CD4+ and CD8+ T cell frequencies following BCG revaccination at 2 time points (Extended Data Fig. 7): first, at 8–10 weeks post BCG revaccination (T2), where a significant increase in the frequencies of BCG-specific IFN-γ or IL-2 CD4+ but not CD8+ T cells were noted in the BCG-RV but not BCG-NRV group, relative to paired baseline samples (T0) (Extended Data Fig. 7A and 7B). *M. tuberculosis* specific recombinant ESAT-6/CFP10 fusion protein served as a negative control^56^; as BCG lacks this region. No change between time points in all samples tested was noted to ESAT-6/CFP10 stimulation (Extended Data Fig. 7B). Second, we demonstrate that higher BCG-specific CD4+ T cell responses noted in BCG-RV at 8–10 weeks post BCG vaccination persist until the T6 time point of the study, which is 78–94 weeks post BCG revaccination (Extended Data Fig. 7C and see Figure 1), with no change in MTb-specific CD8 T cell frequencies between BCG-RV and BCG-NRV, highlighting BCG vaccination to more robustly induce CD4+ rather than CD8+ T cell effectors at the time points studied.

### BCG revaccination enhances the breadth of the COVISHIELD^™^ induced immune response.

**Figure 5:**
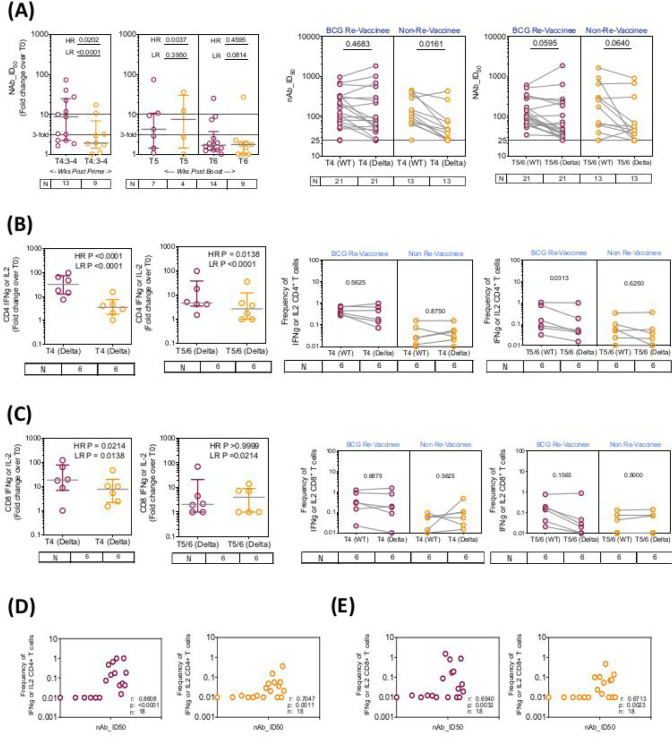
The breadth of the spike response in BCG-RV and BCG-NRV to Wild-type and Delta variant (B1.617.2). **(A)** Comparison of neutralizing antibody responses (NAb ID_50_) to the Delta variant (B1.617.2) in samples collected from COVISHIELD^™^ vaccinated BCG-RV (purple circles) and BCG-NRV (orange circles) at 3–4 weeks post-prime (T4:3–4), 6–7 weeks post boost (T5) and 20–23 weeks post boost (T6). Grouped scatter plots depict the median (horizontal grey line) and interquartile range of fold change in NAb ID_50_ over baseline. Comparison of neutralizing antibody responses (NAb ID_50_) to wild-type versus Delta variant (B1.617.2) in samples collected from COVISHIELD^™^ vaccinated BCG-RV (purple circles) and BCG-NRV (orange circles) at 2–4 weeks post prime (T4) and 6–7 weeks (T5) or 20–23 weeks (T6) post-boost. **(B)** CD4+ and **(C)** CD8+ T cell responses to the WT and delta variant in COVISHIELD^™^ vaccinated subjects. PBMCs from individuals collected from COVISHIELD^™^ vaccinated BCG-RV (purple circles) and BCG-NRV (orange circles) were stimulated with Spike peptide pool to the delta strain (B1.617.2) and its matched reference WT (0.06 nM) for 20 hr. CD4+ and CD8+ T cells were analyzed for intracellular expression of IFN-γ or IL-2. Comparison of the frequencies of IFN-γ or IL-2 in (B) CD4+ and (C) CD8+ T cells to the Delta at post-prime (T4) and post boost (T5/6). Grouped scatter plots depict the median (horizontal grey line) and interquartile range of fold change in frequencies of IFN-γ or IL-2 over baseline. Comparison of the frequencies of IFN-γ or IL-2 in (B) CD4+ and (C) CD8+ T cells to Delta variant (B1.617.2) and the matched WT reference spike pool in samples collected from COVISHIELD^™^ vaccinated BCG-RV (purple circles) and BCG-NRV (orange circles) at post prime (T4) and post-boost (T5/6). **(D and E)** Correlations between IFN-γ or IL-2 expression in CD4+ (left panel) and CD8+ (right panel) T cells and corresponding neutralizing antibody responses (NAb ID_50_) to the delta variant in COVISHIELD^™^ vaccinated BCG-RV (purple circles) and BCG-NRV (orange circles) measured overtime. Kruskal-Wallis test with Dunn’s correction was used for determining statistical significance between longitudinal timepoints in (A). The proportion of each group that showed positive neutralizing antibody titres and IFN-γ or IL-2 CD4+ and CD8+ T cell response to Spike in (A) and (C) were compared between COVISHIELD^™^ vaccinated BCG-RV (purple circles) and BCG-NRV (orange circles) by using Fisher’s exact test. HR indicates the p-value for high-responders in each group (subjects with >10-fold change for NAb, CD4+ or CD8+ T-cell responses). LR indicates the p-value for low-responders (>3-fold change for NAb and >4-fold change for CD4+ or CD8+ T-cell responses). Wilcoxon matched paired t-test was used for determining statistical significance in (B) and (D).

We next determined the efficiency with which COVISHIELD^™^ vaccine induced NAb and T cells recognise the spike protein of the Delta variant (B1.617.2). Extended Data Fig. 8A (line graphs) show that while both BCG-RV and BCG-NRV induce significant NAb response after prime, there is a sharp decline of Delta-specific NAb to near baseline across several donors at 20–23 weeks post-booster, implying that Delta-specific Abs were not as well sustained as NAb to the wild-type (WT) SARS-CoV-2 Wuhan strain (see Fig 2). The differences between groups comparing fold induction of responses over matched baseline values (Fig. 5A scatter graphs) were striking: the BCG-RV group had a significantly higher proportion of high and low responders after prime, but these differences were not sustained after the booster with few high responders detected at 20–23 weeks post booster (Fig. 5A scatter graphs). This was also confirmed using a Wilcoxon paired t-test analysis of the NAb response to WT versus Delta strains in each subject (Fig. 5A), which showed that after prime the BCG-RV group had equally efficient NAb to both strains, whereas BCG-NRV had significantly lower NAb to Delta. After the booster, there was a trend for subjects in both groups to have higher NAb to WT, but this difference did not reach significance. These data highlight priming alone induces a more efficient NAb to both WT and Delta in BCG-RV compared to BCG-NRV.

Matched analysis of the breadth of CD4+ and CD8+ T cell responses is shown in Fig. 5B and 5C respectively to peptides spanning the Delta mutation relative to matched epitopes in the Wuhan strain in a subset of 6 subjects. Extended Data Fig. 8B and 8C (line graphs) show that frequencies of IFN-γ or IL-2 expressing CD4+ and CD8+ T cells are enhanced in response to delta strain in both BCG-RV and BCG-NRV after prime, although the extent of induced response was considerably lower in BCG-NRV and waned substantially by 20–23 weeks post-booster in both groups. Fig. 5B and 5C show BCG-RV comprised significantly higher proportion of both CD4 and CD8 high and low responders respectively, highlighting BCG-NRV to be overall weaker T-cell responders to Delta SARS-CoV-2. This was also confirmed using Wilcoxon paired t-test analysis, which showed equally efficient induction of CD4 and CD8 T cell responses to both strains within a given donor at prime and a decline of Delta responses at 20–23 weeks post boost (Fig. 5B and 5C). Figure 5D and 5E confirm that Delta-specific NAb and CD4+ or CD8+ T cell responses correlated significantly. Collectively, above data highlight that BCG-RV mount more robust spike-specific NAb and CD4+ and CD8+ T cell responses to the parent Wuhan and Delta strains compared to BCG-NRV.

### BCG revaccination boosts monocyte and PBMC PAMP-induced effector cytokines that are associated with trained immunity.

**Figure 6:**
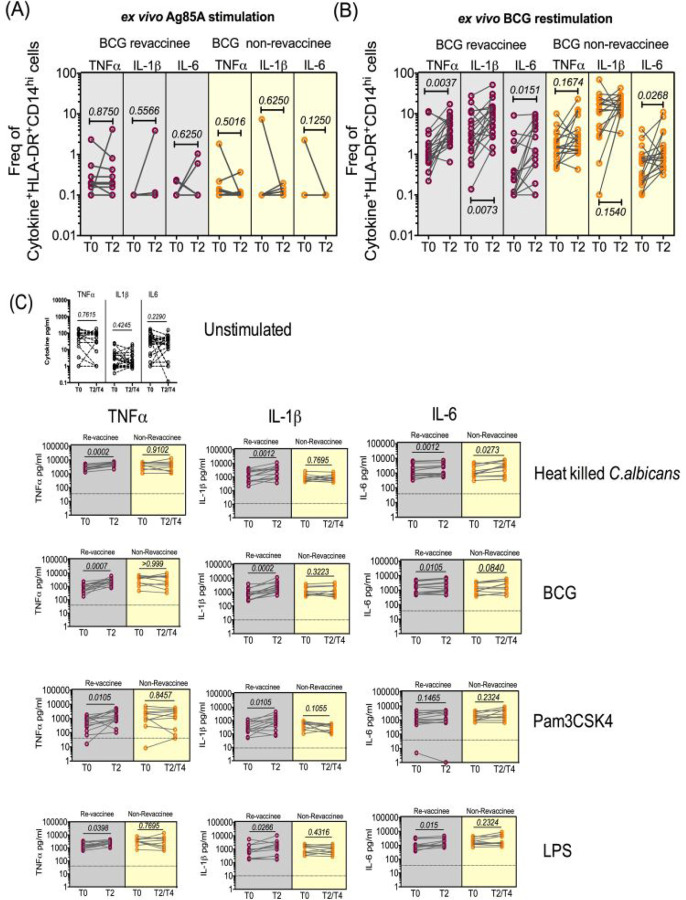
BCG revaccination boosts innate effector responses in HLA-DR+ monocytes and trained immunity effectors to PAMP stimulation. Whole blood from 20 BCG-RV and 18 BCG-NRV at baseline (T0) and 10–12 weeks (T2) post-revaccination was stimulated or not with either Ag85A **(A)** or BCG **(B)** for 12 hrs after which samples were subjected to RBC lysis, fixed, frozen and archived. Frozen samples were thawed, washed and stained with a 17-color antibody panel to assess expression of innate effectors TNF-α, IL-1β and IL-6 in the monocyte compartment. Frequencies of TNF-α+, IL-1β+ and IL-6+ monocytes after background subtraction were plotted for comparison of responses at T0 and T2. Grey shaded areas of the graphs show data for BCG-RV and yellow shaded areas for BCG-NRV. Wilcoxon signed-rank t-test was used for determining statistical significance. **(C)** Levels of TNF-α, IL-1β and IL-6 upon PBMC restimulation. PBMC from 13 BCG-RV and 10 BCG-NRV at baseline (T0), 10–12 weeks (T2) or 51–68 weeks (T4) post-re-vaccination were stimulated or not with 10^6^ cfu/ml heat-inactivated *C. albicans*, 0.2×10^6^ cfu/ml BCG, 1ng/ml LPS and 50μg/ml Pam_3_CSK_4_ for 24 hr after which supernatants were collected and ELISA for TNF-α, IL-1β and IL-6. Absolute concentrations of secreted cytokines were read off a standard curve and plotted after subtraction of background. Cytokines secreted by unstimulated cells (i.e., background) are shown separately at the top of the figure. Grey shaded areas of the graphs show data for BCG-RV and yellow shaded areas for BCG-NRV. Wilcoxon signed-rank t-test was used for determining statistical significance.

One acknowledged mechanism through which BCG potentially boosts immune responses to a heterologous vaccine is by augmenting monocyte and PBMC PAMP-stimulated responses including expression of TNF-α, IL-1β and IL-6 implicated in trained immunity. We therefore tested monocyte and PBMC responses to PAMP stimulation in the same subjects probed for COVISHIELD^™^ induced vaccine responses. Samples archived prior to the COVID-19 pandemic were tested to mitigate against the potential of either the COVISHIELD^™^ vaccine or exposure to SARS-CoV-2 to induce PAMP responses. We compared pre-pandemic baseline (T0) with samples harvested 8–10 weeks post BCG revaccination (T2, see Fig. 1) with matched BCG-NRV samples. Figure 6 first shows the frequency of HLA-DR+CD14+ monocyte responses following *in vitro* stimulation with BCG (a recognised PAMP) in a flow cytometry assay (representative staining shown in Extended Data Fig. 9). Parallel cultures with Mtb Antigen85A (Ag85A) T cell epitope peptides served as a negative control with Ag85A predicted to not stimulate monocytes efficiently (Fig. 6A). The frequency of HLA-DR+ TNF-α and IL-1β expressing CD14+ cells was significantly higher at 8–10 weeks post BCG revaccination (T2) relative to baseline (T0) in BCG-RV but not BCG-NRV samples (Fig. 6B). Significant fluctuation of IL-6+ CD14+ monocytes was noted in BCG-NRV longitudinal samples thereby precluding the identification of specific changes in BCG-induced IL-6+ monocytes. No significant longitudinal changes in all three effectors were noted to Ag85A stimulation, highlighting specificity of BCG induced TNF-α and IL-1β expression(Fig. 6A). Next, we probed PAMP responses in matched PBMC in a 24hr cytokine secretion assay (Fig. 6C). The data show TNF-α and IL-1β secretion following stimulation with BCG, *Candida albicans,* Pam_3_CSK_4_ and LPS to be significantly higher at T2 compared to T0 in BCG-RV but not BCG-NRV cultures (Fig. 6C). Consistent with the flow cytometry data (Fig. 6A and 6B), the induction of IL-6 was weaker and fluctuated in BCG-NRV. These data confirm that BCG revaccination can augment innate responses at the monocyte and PBMC level; these early innate changes may serve as a potential mechanism in boosting subsequent COVISHIELD^™^ vaccine induced immune responses in the same subjects.

## DISCUSSION

This study provides the first in-depth analysis of immune responses to COVISHIELD^™^, the most widely distributed COVID19 vaccine in India combined with novel insights into the beneficial adjuvant effects of prior BCG vaccination on subsequent COVISHIELD^™^ induced immune responses; our study is distinct from ongoing clinical trials testing impact of BCG vaccination on SARS-CoV-2 infection outcome in a COVID-19 unvaccinated population. Three important and unique strengths support our conclusions: (i) the inclusion of a control group: we were able to compare the COVISHIELD^™^ vaccine induced Ab and T cell responses in both BCG-RV and BCG-NRV similar for age, BCG vaccination at birth and time post COVISHIELD^™^ vaccination (ii) the ability to probe COVISHIELD^™^ vaccine induced responses without interference of infection: all subjects included were seronegative with no history of SARS-CoV-2 infection; further, sampling after COVISHIELD^™^ prime was uniformly before the widespread second COVID-19 wave in India, also reflected in COVISHIELD^™^ immune response kinetics being similar to previous studies in seronegative populations ^51–55^. (iii) Importantly, the access to pre-pandemic, pre-BCG and pre-COVISHIELD^™^ vaccine samples enabled longitudinal comparison of vaccine induced responses relative to an unequivocal baseline reading for each immune assay, thereby establishing unambiguous immune assay cut-off values.

The two principal immunological mechanisms by which COVISHIELD^™^ mediates protection against COVID-19 disease severity is through induction of SARS-CoV-2 spike-specific NAbs and T cells^57^. We demonstrate that BCG does not significantly alter the published kinetics of spike-specific Ab and T cell responses induced by the COVISHIELD^™^ vaccine in a seronegative population, with Ab binding and NAb levels peaking 3 weeks post prime (as recorded in Fig. 2) and further enhanced at 2 weeks post boost^51–55, 58–60^, a time point that we were unable to collect. However, we show a clear boost to Ab binding and NAb levels at 5–6 weeks, implying the booster dose effect is sustained up to 5–6 weeks before waning. We show frequencies of spike-specific IFN-γ or IL-2 CD4+ and CD8+ T cells rising significantly over baseline at 2 weeks and continuing to rise over 3 and 4 weeks post prime (Fig. 2). However, unlike Ab responses, our data, as reported^51–55^, show no major booster effect of the COVISHIELD^™^ vaccine induced T cell responses (Fig. 2).

Whilst BCG did not alter the kinetics of the COVISHIELD^™^ response, there was significant impact on its quality in three major ways: First, Ab and T cell responses were significantly higher in BCG-RV, including a greater proportion of high responders based on fold induction of the measured immune response over baseline. Some BCG-RV individuals had exceptionally high T cell responses (>10-fold change) that persisted till 20–23 weeks post-boost; such high responses were not detected or declined sharply in BCG-NRV. This heterogeneity may be intrinsic to the COVISHIELD^™^ vaccine^61^ and potentially amplified by BCG. BCG revaccination therefore synergizes with COVISHIELD^™^ to amplify vaccine-specific Ab and T cell responses as well as enhance the durability of the induced immune response. Second, BCG helped induce a more polyfunctional T cell response, a characteristic that ascertains vaccine efficacy against chronic viral infections^62^, including SARS-CoV-2 where vaccine-induced multifunctional T cells correlate with enhanced protection from emerging variants^63^. Interestingly, one study showed polyfunctional T cells to be enhanced following the ChAdOx1 nCoV-19 vaccine booster indicating the booster may serve to enhance the quality and not just magnitude of a vaccine-induced response^63^. We contend that significant induction of polyfunctional spike-specific T cells and their persistence after the booster can potentially contribute to the heterologous benefit of BCG. Third, BCG-RV produced a more robust response to the Delta mutant of SARS-CoV-2 highlighting greater breadth of immune responses, a function that was globally, including India, associated with milder disease in AstraZeneca vaccinees during second wave of the SARS-CoV-2 pandemic^64–66^. With a strong correlation noted between NAb and T cells specific for both the Wuhan and Delta strains, we contend that BCG vaccination has the potential to expand the breadth of the Ab and T cell response against SARS-CoV-2 variants of concern.

Our data is consistent with previous work highlighting the benefit of prior or synchronous BCG vaccination in boosting heterologous vaccine responses^67^, including to a trivalent influenza vaccine^44^ and to vaccination against pneumococcus, tetanus toxoid, measles, mumps, diphtheria and polio in BCG immunized infants^46–48^. Our data is also consistent with the results of the Mexican study which demonstrated prior BCG vaccination to enhance the Pfizer/Biotech induced NAb response,4 weeks after BCG vaccination^49^. In the context of COVID-19, BCG may not be unique and is consistent with emerging acceptance of the benefits of heterologous vaccination strategies. It’s been noted that immunization with existing vaccines such as the Influenza, OPV, MMR, Varicella Zoster in the recent past (≤ 5 years) can confer protection against SARS-CoV-2 by reducing infection rates, improving clinical outcomes and /or boosting NAbs induced during infection^68^. Indeed, heterologous prime-boost immunization regimens *per se* maybe more beneficial, i.e., ChAdOx1 nCoV-19 and mRNA-1273 or ChAdOx1 nCoV-19 and BioNTech have been shown to augment COVID-19 vaccine efficacy by enhancing spike-specific IgG, NAbs as well as CD4+ and CD8+ T cells including robust recognition of variants of concern above levels induced by homologous vaccination^69–73^.

One important mechanism by which BCG vaccination can boost heterologous vaccine responses is its intrinsic PAMP characteristics and ability to regulate innate immunity. Individuals in our study who showed boosted adaptive responses to COVISHIELD^™^ also exhibited evidence of trained immunity 8–12 weeks post BCG revaccination (Fig. 6) in terms of enhanced TNF-α and IL-1β secretion upon *in vitro* PAMP restimulation. Previously published studies in animals and humans show enhanced adaptive responses often follow induction of BCG induced trained immunity suggesting that TI can indeed impact the adaptive arm of the immune system^8,44,74^. Mice immunised with BCG were found to be resistant to subsequent vaccinia virus infection and this was mediated via the CD4+ T cell response^74^. Similarly higher Th1 and Th17 cytokine levels in addition to innate responses were observed to *in vitro* stimulation with *Staphylococcus aureus* and *Candida albicans* in PBMC from individuals vaccinated with BCG^8^. Also, volunteers who received BCG prior to influenza vaccination had signatures of trained immunity as well as augmented anti-H1N1 humoral responses^44^.

Our observation that the heterologous benefit of BCG was more evident on COVISHIELD^™^ induced spike-specific CD4+ rather than CD8+ T cells is consistent with BCG as a recognised inducer of Th1 CD4+ T cell effectors through three potential mechanisms: firstly, trained monocytes have higher expression of MHC-II and costimulatory molecules CD80 and CD86; thus making them better antigen presenting cells for CD4+ T cell activation^75,76^; secondly, trained monocytes have a higher expression of PRRs like CD14, TLR4 and mannose receptor and produce more pro-inflammatory cytokines such as TNF-α and IL-1β which can enhance T cell responses^8^ and thirdly, cytokines secreted by trained monocytes e.g., IL-1β and IL-6 are key drivers of Th differentiation to Th1, Th17 or ex-Th17- sub-sets that have been shown to be correlates of protection against viral and bacterial infections^77–79^. Apart from these suggested mechanisms, it has been speculated that BCG vaccination might lower thresholds for T cell activation on account of the cytokine milieu that exists due to primed/trained monocytes^80^. It should be noted that boosted T cell responses to COVISHIELD^™^ in individuals vaccinated with BCG might also be due to presence of cross-reactive epitopes in BCG and SARS-CoV-2 vaccines^81^.

Our data has important healthcare implications despite a small sample size imposed by COVID-19 lockdown restrictions. Whilst ongoing global studies are testing BCG impact on SARS-CoV-2 infection outcome, our study was designed to test whether immune responses induced by an otherwise highly efficacious and the most widely distributed vaccine in India, namely COVISHIELD^™^, can be further boosted or enhanced in a SARS-CoV-2 infection and vaccine naïve population. The fact that BCG does have this potential in a young healthy population calls for further analysis on timing/ dose/ nature of prior BCG vaccination on heterologous vaccine responses in the elderly and the immunocompromised versus testing if pre-existing COVID-19 vaccine or SARS-CoV-2 infection induced responses can be enhanced by subsequent BCG vaccination. We highlight the potential of using a cheap and globally available vaccine as an adjuvant for novel and emerging vaccines, an area of significant scientific interest^82,83^, with the added advantage that the timeline over which BCG adjuvant effects have been noted span several years^44,47^. We postulate this to be linked to BCG leaving an imprint on innate cells/responses combined with its ability to induce long lasting mycobacterial antigen specific CD4+ memory T-cells which can provide T-T and T-B cell benefit, a concept highlighted by 1980’s work showing Ab responses induced by a foreign antigen coupled to tuberculin being significantly higher in BCG pre-sensitized animals^84^. We call for further studies to understand heterologous benefits of BCG and the associated impact of tuberculosis prevalence on COVID-19 vaccine immunity.

## METHODS

### Ethics Statement.

This study was performed in accordance with the relevant guidelines and regulations stated in the Declaration of Helsinki and was approved by the Institutional Ethics Ethical Review Committee of St. John Medical College Hospital, Bangalore, IEC Ref no: (IEC/1/896/2018).

### Study participants.

Apparent healthy adolescent male and female healthcare workers of St. John’s Medical College-Hospital, Bangalore, India were invited to participate in this prospective observational study from October 2019 to June 2021. All potential participants underwent a screening criteria and subjects with chronic illness such as hypertension, diabetes mellitus, heart disease, cancer, kidney / thyroid illness, asthma, epilepsy, jaundice or with a history of clinical tuberculosis disease and on medication were excluded from the study. All included subjects were assigned a unique serial number and baseline information such as age, gender, medical history, occupation, vaccination status and family history pertaining to tuberculosis was obtained. All participants were BCG-vaccinated at birth. Basic anthropometry measurements such as height (cm), weight (kg) using standard validated and calibrated instruments were used, and body mass index (kg/m^2^) was computed. Relevant clinical information of study participants was documented in a proforma and is summarized in Table 1 and detailed follow-up questionnaire is provided in Supplemental File 1. Blood from study participants was screened for Mtb infection by the standard QFT TB Gold In-tube test (Qiagen) performed at Department of Microbiology, SJMCH, India, and were classified as either IGRA+ or IGRA− of which 66 IGRA− subjects were enrolled for the study (Fig. 1A).

### Study design.

A prospective observational study was conducted to evaluate the effect of BCG revaccination on subsequent anti-SARS-CoV-2 vaccination. The volunteers were given the choice of either being BCG re-vaccinated (BCG-RV, Group 1) or not (BCG-NRV, Group 2) and continue the study protocol of follow-up until 1.6 years. BCG vaccine (TUBERVAC^™^, Russian BCG strain manufactured at Serum Institute of India, Pune, India), used widely in the Indian national immunization program, was administered intradermally at day 0 at an adult dose of 2 × 10^5^ to 8 × 10^5^ CFU in participants from BCG-RV (n = 35) and BCG-NRV (n = 31) subjects who were not BCG revaccinated served as control. Blood was collected from participants at days 0 (T0) and day 1 (T1), and at weeks 10–12 weeks (T2), 51–68 weeks (T4), 64–77 weeks (T5) and 78–94 weeks (T6) after BCG vaccination (Fig. 1B). Some vaccinees, after BCG vaccination, reported minor side effects, which included itching, rash, or pain at the site of vaccination; mild fever; cough; and headache (Supplemental File 1). All participants received two doses of the COVISHIELD^™^ SARS-CoV-2 vaccine, 7–8 weeks apart, and blood was collected 2–4 weeks post-prime (T4) and either 6–7 weeks (T5) or 20–24 weeks (T6) post-boost (Fig. 1B). No serious side effects were reported and none of the participants become active TB+ during the entire duration of study. Individuals who turned COVID RT-PCR positive were excluded from downstream assays.

### Peripheral blood mononuclear cell isolation.

Anticoagulated blood (total 16–20 ml) was collected in Na-Heparin tubes (BD, Franklin Lakes NJ, USA) and peripheral blood mononuclear cells (PBMCs) were isolated using 15ml ACCUSPIN (Sigma-Aldrich) tubes by density centrifugation following manufacturer’s instructions. Blood was diluted two-fold with PBS (Gibco by Life Technologies, Washington, DC, USA) + 2% FBS (Gibco), pipetted into ACCUSPIN tubes prefilled with Histopaque 1077 (Sigma) and centrifuged at 1000g for 15 minutes at room temperature without deceleration. PBMCs from the buffy coat were washed twice with PBS + 2% FBS, then re-suspended at 10 × 10^6^ cells/mL in cryopreservation medium (90% FBS and 10% DMSO), incubated overnight at −80°C (in Mr. Frosty™ freezing container; Nalgene, Rochester, New York, U.S.) and were stored in liquid nitrogen until further analyses.

### ICS assay for whole blood stimulation assay and multiparameter flow cytometry.

Heparinized whole blood was collected from participants and processed within 30–45 min of phlebotomy, as previously described^85^. Briefly, 400 μl of blood was pipetted into 5ml polypropylene tubes (Sarstedt, Germany) and stimulated with Ag85A peptide pools (1 μg/ml per peptide), BCG (0.2 × 10^6^ CFU/ml), or purified recombinant protein ESAT-6/CFP10 (10μg/ml) together with anti-CD28/CD49d costimulatory mAbs at 0.5 μg/ml. Culture medium with anti-CD28/CD49d was used as unstimulated negative control. Blood was incubated at 37°C for a total of 12 hr, and Brefeldin A + Monensin (Biolegend) diluted to a final concentration of 1X from a 1000X stock was added in the final 10 hr of stimulation. After stimulation, blood was treated with 2 μM EDTA, RBCs were lysed with 4.5 ml 1X FACS Lysing solution (BD), and fixed white blood cells were transferred to liquid nitrogen in freezing medium containing 10% DMSO, 40% FCS, and 50% RPMI 1640. On the day of staining, cryopreserved whole-blood samples were thawed in a water bath at 37°C for 2 min. Thawed cells were transferred to labelled tubes containing 2 mL of PBS and were centrifuged at 800g for 5 min. Cells were then stained with a 50 μl cell surface staining cocktail prepared in PBS + 2% FBS for 30 min at room temperature (RT) in the dark. Next, the cells were washed with PBS, permeabilized by adding 200μl 1X Perm/Wash solution (BD Biosciences) and incubated at RT for 20 min. Pelleted cells were immediately stained with a 50 μl cocktail containing antibodies against intracellular markers for 30 min at RT. Cells were washed and re-suspended in 100μl of 1% paraformaldehyde (Electron Microscopy Sciences, Hatfield, PA, USA) for flow cytometry analysis. Please refer Table 2 for details of antibodies used for staining.

### ICS assay with SARS-CoV-2 specific peptides.

Flow cytometry was used to examine SARS-CoV-2-specific CD4+ and CD8+ T cell responses using a validated ICS assay^50^. Briefly, cryopreserved peripheral blood mononuclear cells (PBMC) were thawed, and seeded in 96-well round-bottom plates (Costar) at a density of 1 × 10^6^ cells/well in complete RPMI medium after 2hr of rest. Next, 1 μg/ml FastImmune CD28/CD49d (BD Biosciences) was added to all the wells. Cells were then stimulated for 20hr at 37°C with peptide pools spanning the entire sequence of SARS-CoV-2 structural proteins, i.e., spike (S), nucleocapsid (N) or membrane (M) at a final concentration of 0.06nM. In few experiments, cells were also stimulated with the Delta variant (B.1.617.2) and the matched wild-type peptide pool. [The PepTivator SARS-CoV-2 Prot_S B.1.617.2 Mutation Pool covers selectively the mutated regions in the surface or spike glycoprotein (“S”) of the SARS-CoV-2 B.1.617.2 lineage (Delta variant), one of the subvariants of B.1.617. PepTivator SARS-CoV-2 Prot_S B.1.617.2 WT Reference Pool consists of the 32 homologous peptides of the Wuhan sequence and serves as a control.] SARS-CoV-2 PepTivator (Miltenyi Biotec) peptide pools are comprised of peptides that are 15 amino acids long and overlap by 11 amino acids. For negative control, cells were incubated with an amount of sterile water equivalent to that present in peptide-stimulated samples. CEFT peptide pool (JPT Peptide Technologies) at a concentration of 1 μg/ml was included as a common recall antigen whereas Phytohemagglutinin (PHA, Remel) at a concentration of 4 μg/ml was included as the positive control. Brefeldin A and Monensin (1X, BioLegend) were included for the last 18 hr of stimulation to prevent cytokine release. Next day, PBMCs were first stained with Live/Dead fixable Aqua dead cell stain or AviD (Invitrogen) to exclude dead cells from analysis for 10 min at room temperature (RT). Cells were then washed and stained with antibodies to cell surface markers. Cells were then fixed with 1X FACS lysis buffer (BD Biosciences) for 10 min and permeabilized with 1X BD Perm/Wash buffer (BD Biosciences) for 20 min. Cells were then incubated with a cocktail containing antibodies to intracellular cytokines and activation-induced markers (AIM). Antibody incubations were all performed at RT for 30 min in the dark. Cells were washed, resuspended in PBS containing 0.4% BSA and kept at 4°C until acquisition. Please refer Table 3 for details of antibodies used for staining.

### Flow cytometry data analysis.

Cell fluorescence were acquired on the 5-laser, 18-parameter BD FACSAria™ Fusion flow cytometer (BD Biosciences, San Jose, CA) using BD FACSDiva™ version 8.0.1 software. Cytometer Setting and Tracking (CST) beads (BD Biosciences) were acquired before each experiment to ensure that cytometer parameters remained consistent across all experiments. Negative and single-stained compensation beads (eBioscience) were acquired for each experiment, before sample acquisition, and used to calculate the compensation matrix. Samples were analyzed using FlowJo 10.8.0 (BD Biosciences). Gating strategy can be found in Extended Data Fig. 2A. Representative flow plots for cytokine gating are shown in Extended Data Fig. 2B. All antigen-specific cytokine frequencies are reported after background subtraction of identical gates from the same sample incubated with negative control stimulation. Samples were invalidated if <20,000 live CD3+ T cells were collected. Similarly, the total number of CD4+ T cells must have exceeded 10,000 and the total number of CD8+ T cells must have exceeded 5,000 for the assay data to be included in the analysis. Cells expressing IFN-γ and/or IL-2 was the primary immunogenicity endpoint for CD4+ and CD8+ T cells with an assay cut-off ≥0.02% based on staining of pre-pandemic samples. An individual was considered a responder post COVISHIELD^™^ prime and booster dose, if the frequency of IFN-γ and/or IL-2 expressing CD4+ or CD8+ T cells upon stimulation with the individual SARS-CoV-2 peptide pools was minimum 2-fold higher than baseline. Background subtractions were performed in Pestle version 1.8. Polyfunctionality of the stimulated cells was analysed with SPICE version 6.1 software^86^ as described previously. Briefly, Boolean analysis was performed to define the multifunctional profiles on FlowJo 10.8.0. The analysis included IFN-γ, IL-2, and TNF-α gated on CD4+ cells and CD8+ cells. The overall response to spike was defined as the sum of the background subtracted responses to each combination of individual cytokines.

### Innate PAMP (Pathogen associated molecular patterns) stimulation of PBMC.

Cryopreserved PBMC samples were rapidly thawed in a 37°C water bath, transferred to 15 ml tubes containing ~ 3 ml RPMI+10% FBS and centrifuged at 800g for 5 min at RT. A total of 200000 cells resuspended in 200 μL culture medium [RPMI-1640 (GIBCO, Invitrogen) supplemented with 10% FBS (GIBCO), 100 U/ml penicillin and 100 μg/ml streptomycin, (SIGMA)] were seeded per well in 96-well flat-bottom plates (Eppendorf) and stimulated with either 10^6^ cfu/ml of heat-killed *Candida albicans* Strain SC5314 (kind gift from David Moyes; King’s College London), 0.2 × 10^6^ cfu/ml of BCG (TUBERVAC, Serum Institute of India), 50 μg/ml Pam_3_CSK_4_ (Sigma) or 1ng/ml LPS (Sigma). Cells cultured with medium alone were used as negative control. Cells were cultured for 24 hr after which plates were centrifuged at 800g for 3 minutes and culture supernatants were collected and frozen at −20°C till ELISA was performed.

### ELISA measurements for TNF-α, IL-1β and IL-6.

Supernatants from PAMP stimulation cultures were used for measuring levels of TNF-α, IL-1β and IL-6. Commercial ELISA kits (TNFα-BD; IL-1β-Biolegend and IL-6-Biolegend) were used and ELISA was performed according to the manufacturer’s instructions. A standard sandwich ELISA was performed in each case using a separate coating and detection antibody. Bound chemokine was detected finally by the enzymatic conversion of 3,3’,5,5’ trimethylbenzidine or TMB (Sigma) by HRP conjugated to the secondary antibody. Colour was read at 450 nm using VERSA Max Microplate Reader (Molecular Devices). Absolute concentration of cytokine was calculated using a standard curve. The linear range of detection for TNF-α was 40 – 2500 pg/ml, for IL-1β was 10 – 1250 pg/ml and for IL-6 it was 40 – 5000 pg/ml. For each ELISA, assay background was subtracted from absorbance values. Also, the spontaneous cytokine release in cells cultured with medium alone was subtracted from all PAMP stimulation conditions.

### Recombinant full-length Spike, RBD and Nucleocapsid proteins.

Recombinant Spike and RBD for ELISA were expressed and purified as previously described^87^. S protein consists of a pre-fusion S ectodomain residues 1–1138 with proline substitutions at amino acid positions 986 and 987, a GGGG substitution at the furin cleavage site (amino acids 682–685) and an N terminal T4 trimerisation domain followed by a Strep-tag II^88^. The plasmid was obtained from Philip Brouwer, Marit van Gils and Rogier Sanders at The University of Amsterdam. The protein was expressed in 1 L HEK-293F cells (Invitrogen) grown in suspension at a density of 1.5 million cells/mL. The culture was transfected with 325 μg of DNA using PEI-Max (1 mg/mL, Polysciences) at a 1:3 ratio. Supernatant was harvested after 7 days and purified using StrepTactinXT Superflow high capacity 50% suspension according to the manufacturer’s protocol by gravity flow (IBA Life Sciences). The RBD plasmid was obtained from Florian Krammer at Mount Sinai University^89^. Here the natural N-terminal signal peptide of S is fused to the RBD sequence (319 to 541) and joined to a C-terminal hexa-histidine tag. This protein was expressed in 500 mL HEK-293F cells (Invitrogen) at a density of 1.5 million cells/mL. The culture was transfected with 1000 μg of DNA using PEI-Max (1 mg/mL, Polysciences) at a 1:3 ratio. Supernatant was harvested after 7 days and purified using Ni-NTA (Nickel-Nitrilotriacetic acid) agarose beads. N protein was obtained from Leo James and Jakub Luptak at LMB, Cambridge. The N protein used is a truncated construct of the SARS-CoV-2 N protein comprising residues 48–365 (both ordered domains with the native linker) with an N terminal uncleavable hexa-histidine tag.

### In-house ELISA for anti-Spike, RBD or Nucleocapsid IgG.

Plasma was isolated from heparinized whole blood by centrifugation at 400 g for 10 minutes at room temperature. The supernatant was further centrifuged at 800–1200 g for 10 minutes to obtain clear plasma and stored at −80°C until further use. Plasma was heat-inactivated at 56°C for 30 minutes prior to use. SARS-CoV-2 Spike, RBD or Nucleocapsid specific IgG was measured using an in-house ELISA as previously described^88^. Briefly, Spike/RBD purified as previously described^87^ or Nucleocapsid protein (obtained from Leo James lab) diluted to 3 μg/ml in PBS were used to coat 96-well ELISA plates (Nunc MaxiSorp™ flat-bottom) overnight at 4°C. Plates were washed 5x with PBS + 0.05% Tween-20 or PBS-T (Sigma) and blocked with 5% milk (HiMedia) in PBS-T. Plates were then incubated for 2 hr at room temperature with heat-inactivated test plasma (1:25), positive control antibody for S/RBD (CR3022 at 0.2 μg/mL) or for N (CR3009 at 2 μg/mL), positive control convalescent plasma (at 1:25), healthy pre-pandemic plasma (at 1:25) or blocking buffer only. Following a 5x wash, alkaline phosphatase Affinity Pure goat anti-human IgG, Fcγ fragment specific (Jackson ImmunoResearch) diluted 1:1000 in 5% milk/PBS-T was added and incubated for 1 hour at room temperature. After a 5x wash, pNPP substrate (Sigma) was added and OD (405 nm) measurements were taken at 15–20 minutes. For each ELISA, assay background was subtracted from absorbance values.

### Anti-Spike IgG measured using the LIAISON^®^ SARS-CoV2 TrimericS IgG assay.

The LIAISON® SARS-CoV-2 TrimericS IgG assay^90^ (Diasorin.com, accessed on 31^st^ Jan22) was used to measure the Anti-spike IgG levels in the collected plasma samples. This commercial platform is a new generation chemiluminescence immunoassay, using a recombinant Trimeric Spike protein as the capture antigen and provides results within 35 minutes. The assay has a range of 4.81 BAU/ml to 2080 BAU/ml (Binding Antibody Units/ml). Samples with high titers (>2080 BAU/ml), were diluted further with the diluent provided with the kit as per manufacturer guidelines. The binding antibody units measured in this assay are mapped to the 1^st^ WHO international standard for anti SARS-CoV-2 immunoglobulin (NIBSC Code-20/136). Any sample below 33.8 BAU/ml is reported as negative for Anti-Spike IgG antibody.

### SARS-CoV2 pseudotyped virus preparation.

Pseudotyped HIV virus incorporating the SARS-CoV-2 spike protein [Wuhan-1 and delta (B.1.617.2)] were produced in a 10 cm dish seeded the day prior with 7.5×10^6^ HEK293T/17 cells in 10 ml of complete Dulbecco’s Modified Eagle’s Medium (DMEM-C, 10% FBS and 1% Pen/Strep) containing 10% (vol/vol) foetal bovine serum (FBS), 100 IU/ml penicillin and 100 μg/ml streptomycin. Cells were transfected using 90 μg of PEI-Max (1 mg/mL, Polysciences) with: 15 μg of HIV-luciferase plasmid, 10 μg of HIV 8.91 gag/pol plasmid and 5 μg of SARS-CoV-2 spike protein plasmid^91,92^. The supernatant was harvested 72 hr post-transfection. Pseudotype virus was filtered through a 0.45 μm filter and stored at −80°C until required.

### Viral entry inhibition assay with SARS-CoV2 pseudotyped virus.

Serial dilutions of heat-inactivated plasma samples were prepared with DMEM media (10% FBS and 1% Pen/Strep) and incubated with pseudotype virus for 1-hour at 37°C in 96-well plates. Next, Hela cells stably expressing the ACE2 receptor (provided by Dr James Voss, The Scripps Research Institute) were added (10,000 cells/25 μL per well) and the plates were left for 72 hr. Infection level was assessed in lysed cells with the Bright-Glo luciferase kit (Promega), using a Victor™ X3 multilabel reader (Perkin Elmer). Measurements were performed in duplicate and duplicates used to calculate the ID_50_.

### Statistical analysis:

Data were analysed using FlowJo 10.8.0. Statistical analyses were performed in GraphPad Prism 9.2, unless otherwise stated. The statistical details of the experiments are provided in the respective figure legends. Data plotted in logarithmic scales were expressed as geometric means ± geometric standard deviations (SD). Mann-Whitney U or Wilcoxon signed-rank t tests were applied for unpaired or paired comparisons, respectively. Differences among longitudinal timepoints were evaluated using Kruskal-Wallis and Dunn’s post-test for multiple comparisons. Details pertaining to significance are also noted in the respective legends.

## Extended Data

**EXTENDED DATA Figure 1:** In-House Ab binding assays correlation with NAb. **(A)** Plasma SARS-CoV-2 anti-Spike, RBD and Nucleocapsid (N) protein IgG titres measured by an in-house ELISA in COVISHIELD^™^ vaccinated subjects measured overtime. IgG titres at baseline (T0), 2–4 weeks post-prime (T4), 6–7 weeks (T5) and 20–23 weeks (T6) post-boost. **(B)** Correlations between SARS-CoV-2 anti-Spike, RBD and Nucleocapsid protein IgG titres and corresponding neutralizing antibody responses (NAb ID_50_) in COVISHIELD^™^ vaccinated subjects measured overtime. **(C)** Correlations between SARS-CoV-2 IgG titres measured by LIAISON® SARS-CoV-2 TrimericS IgG assay and corresponding neutralizing antibody responses (NAb ID_50_) in COVISHIELD^™^ vaccinated subjects measured overtime.

**EXTENDED DATA Figure 2:** Representative FACS Gating Strategy. Schematic representation showing sequential gating strategy for analysis of CD4+ and CD8+ T-cells in PBMC. **(A)** Representative flow cytometry plots of spike-specific CD4+ and CD8+ T cells after 20hr stimulation with spike peptide pool compared to negative control (Unstimulated) at T0 (baseline), T4 (post-prime) and T5/6 (post-boost). **(B)** Representative examples from two COVISHIELD^™^ vaccinees.

**EXTENDED DATA Figure 3:** COVID vaccination does not boost responses to SARS-CoV-2 M, N and CEFT. CD4+ and CD8+ T cell responses to SARS-CoV-2 M, N and CEFT in COVISHIELD^™^ vaccinated subjects measured overtime. PBMCs from individuals collected from COVISHIELD^™^ vaccinated BCG-RV (purple circles) and BCG-NRV (orange circles) at baseline (T0), 2–4 weeks post-prime (T4), 6–7 weeks (T5) and 20–23 weeks (T6) post-boost were stimulated with peptide pools specific to SARS-CoV-2 membrane, nucleocapsid proteins (0.06nM) and CEFT (1ug/ml) for 20hr. CD4+ and CD8+ T cells were analyzed for intracellular expression of IFN-γ or IL-2. Line graphs comparing the frequencies of IFN-γ or IL-2 in CD4+ and CD8+ T cells in samples are shown. Kruskal-Wallis test with Dunn’s correction was used for determining statistical significance between longitudinal timepoints.

**EXTENDED DATA Figure 4:** The vaccine induced NAb and T cell responses correlate in BCG-RV and BCG-NRV. Correlations between IFN-γ and/or IL-2 expression in **(A)** CD4+ and **(B)** CD8+ T cells and corresponding neutralizing antibody responses (NAb ID_50_) in COVISHIELD^™^ vaccinated BCG-RV (purple circles) and BCG-NRV (orange circles) measured overtime.

**EXTENDED DATA Figure 5:** SPICE all cytokines: BCG revaccination significantly impacts the quality of the spike-specific CD4+ T cell response in COVISHIELD^™^-vaccinated subjects. Longitudinal multifunctional spike- specific CD4^+^ T cells in COVISHIELD^™^ vaccinees. PBMCs from individuals collected at baseline (BL, red dots), 2–4 weeks post-prime (CSP, blue dots) and 6–7- or 20–23-weeks post-boost (CSB, green dots) were stimulated with spike for 20hr and CD4^+^ T cells were analysed for intracellular expression of 5 cytokines i.e., IFN-γ, IL-2, TNF-α, IL-17 and IL-10 in a standard ICS assay. Boolean gates were created from the individual cytokines (listed above) in FlowJo to divide responding cells into 32 distinct subsets corresponding to all possible combinations of these functions, and the data were analysed using SPICE software. Proportions of multifunctional activity profiles of the spike-specific CD4^+^ T cells from COVISHIELD^™^ vaccinated **(A)** BCG-RV and **(B)** BCG-NRV. The pie charts depict the mean frequency for each of the 7 possible phenotypic profiles of spike-specific CD4+ T cells. Each slice of the pie corresponds to a distinct combination of three cytokines (IFN-γ, IL-2, TNF-α, IL-17 and IL-10) produced in response to spike stimulation. The arcs surrounding the pie charts indicate the proportion of the responses contributed by each of the single cytokines. Keys to the colours used for different cytokines in the pie chart arcs and the categories in the slices are shown. Data were analysed for statistical significance using Wilcoxon signed-rank test. Background subtracted and log data analysed in all cases. P < 0.05 was considered statistically significant.

**EXTENDED DATA Figure 6:** Comparison of the multifunctional activity profiles of the spike-specific CD4+ and CD8+ T cells in BCG-RV and BCG-NRV. Proportions of multifunctional activity profiles of the spike-specific CD4+ and CD8+ T cells from COVISHIELD^™^-vaccinated BCG-RV (purple dots) and BCG-NRV (orange dots) ( **A & C)** post-prime and **(B & D)** post-boost. Data were analyzed for statistical significance using unpaired Student’s *t* test with a Welch’s correction. Background subtracted and log data analyzed in all cases. P < 0.05 was considered statistically significant.

**EXTENDED DATA Figure 7:** BCG revaccination boosts T cell responses to Mtb antigens at 8–10 weeks post vaccination prior to COVIDSHIELD^™^ vaccination. **(A)** A representative gating strategy for obtaining CD4+ and CD8+ T cells is shown. Also shown are representative plots for IFN-γ and IL-2 expression post BCG stimulation at T0 and T2 in a BCG re-vaccinee in CD4+ and CD8+ T cells. **(B)** Mycobacteria specific T cell responses after BCG revaccination. Whole blood from 20 BCG-RV and 9 BCG-NRV at baseline (T0) and 10–12 weeks (T2) post-revaccination was stimulated or not with either BCG or ESAT6/CFP10 with for 12 hrs after which samples were subjected to RBC lysis, fixed, frozen and archived. Frozen samples were thawed, washed and stained with a 17-color antibody panel to assess expression of adaptive effectors IL-2 and IFN-γ in the CD4+ and CD8+ T cells. Frequencies of IFN-γ or IL-2+CD4+ and CD8+ T cells after background subtraction were plotted for comparison of responses at T0 and T2. The upper panel shows data for CD4+ T cells (BCG-RV on the left and BCG-NRV on the right) and lower panel shows data for CD8+ T cells (BCG-RV on the left and BCG-NRV on the right). Wilcoxon signed-rank t-test was used for determining statistical significance. **(C)** PBMCs collected from COVISHIELD^™^ vaccinated BCG-RV (purple circles) and BCG-NRV (orange circles) were stimulated with BCG for 20hr. CD4+ and CD8+ T cells were analyzed for intracellular expression of IFN-γ or IL-2. Grouped scatter plot comparing the frequencies of IFN-γ or IL-2 in CD4+ and CD8+ T cells in samples from COVISHIELD^™^ vaccinated BCG-RV and BCG-NRV (orange circles) collected at 78–94 weeks post BCG re-vaccination and 20–23 weeks post COVISHIELD^™^ boost.

**EXTENDED DATA Figure 8:** The breadth of the spike response in BCG-RV and BCG-NRV to Wild-type and Delta variant (B1.617.2). **(A)** Neutralizing antibody responses (NAb ID_50_) to the SARS-CoV-2 Delta (B1.617.2) variant in COVISHIELD^™^ vaccinated BCG-RV (purple circles) and BCG-NRV (orange circles) measured at baseline (T0), 2-, 3- and 4-weeks post-prime (T4:2, T4:3 and T4:4), 6–7 weeks post boost (T5) and 20–23 weeks post-boost (T6). **(B)** CD4+ and **(C)** CD8+ T cell responses to the delta variant in COVISHIELD^™^ vaccinated subjects. PBMCs from individuals collected from COVISHIELD^™^ vaccinated BCG-RV (purple circles) and BCG-NRV (orange circles) were stimulated with Spike peptide pool to the delta strain (B1.617.2; 0.06 nM) for 20 hr. Frequencies of IFN-γ or IL-2 (B) CD4+ and (C) CD8+ T cells to the Delta measured at baseline (T0), post-prime (T4) and post boost (T5/6) are shown.

**EXTENDED DATA Figure 9:** Representative gating strategies. A representative gating strategy for obtaining HLA-DR+CD14hiCD16− monocytes are shown. Also shown are representative plots for TNF-α, IL-1β and IL-6 expression in the HLA-DR+CD14hiCD16− population in one *ex vivo* unstimulated and BCG stimulated sample from a BCG-RV at T0 and T2.

## Supplementary Material

Supplement 1
